# Prognostic Factors in Idiopathic Sudden Sensorineural Hearing Loss: The Experience of Two Audiology Tertiary Referral Centres

**DOI:** 10.3390/medicina60071130

**Published:** 2024-07-13

**Authors:** Valeria Caragli, Leonardo Franz, Alessandro Incognito, Salvatore Bitonti, Maria Guarnaccia, Roberta Cenedese, Debora Cocimano, Aaron Romano, Giuseppe Canova, Paolo Zanatta, Elisabetta Genovese, Cosimo de Filippis, Gino Marioni

**Affiliations:** 1 Otorhinolaryngology Unit, Department of Medical and Surgical Sciences for Children and Adults, University of Modena and Reggio Emilia, 41121 Modena, Italy; valeria.caragli2017@gmail.com (V.C.); maria.guarnaccia76@gmail.com (M.G.); elisabetta.genovese@unimore.it (E.G.); 2 Phoniatrics and Audiology Unit, Department of Neuroscience DNS, University of Padova, 31100 Treviso, Italy; leonardo.franz@unipd.it (L.F.); alessandro.incognito@aulss2.veneto.it (A.I.); salvatore.bitonti.rm@gmail.com (S.B.); robertacenedese@gmail.com (R.C.); debora.cocimano@studenti.unipd.it (D.C.); aaron.romano@studenti.unipd.it (A.R.); cosimo.defilippis@unipd.it (C.d.F.); 3 Department of Neurosurgery, Treviso Hospital, 31100 Treviso, Italy; giuseppe.canova@aulss2.veneto.it; 4 Department of Anesthesiology and Critical Care, Treviso Hospital, 31100 Treviso, Italy; paolo.zanatta1@aulss2.veneto.it

**Keywords:** sudden, sensorineural hearing loss, idiopathic, prognosis, treatment

## Abstract

*Background and Objectives*: Although different hypotheses have been proposed over time, there is a dearth of information on factors able to predict the response to treatment for idiopathic sudden sensorineural hearing loss (ISSNHL) and hearing recovery. The aim of this study was to apply univariate and multivariate statistical models in a retrospective clinical setting of patients given therapy for ISSNHL at our tertiary academic audiological centers to investigate the prognostic value of clinical signs, symptoms, and comorbidities in relation to hearing recovery. *Materials and Methods*: The inclusion criteria were: history of ISSNHL diagnosed and treated at the Padova or Modena tertiary academic audiological centers; age ≥ 18 years; availability of clinical and audiological outcome data. The exclusion criteria were: hearing loss in acoustic schwannoma, endolymphatic hydrops, meningitis, trauma (head trauma, temporal bone fracture, acoustic trauma), barotrauma, perilymphatic fistula; exposure to noise levels ≥ 80 dB in the work environment; any unilateral or bilateral hearing loss (except for presbycusis) prior to ISSNHL diagnosis; any disorders affecting the external or middle ear; any previous ear surgery; refusal to make medical data available for research purposes. Eighty-six consecutive patients (38 females, 48 males; median age: 58 years; interquartile range: 47.00–69.00 years) were included. A systemic steroid therapy was administered to all patients, either orally with prednisone or intravenously with methylprednisolone. Second-line therapy included intratympanic steroid injections and/or hyperbaric oxygen therapy. *Results*: A multivariate logistic regression model was used, including the non-multicollinear clinical and audiological variables, which showed a *p*-value < 0.10 at the univariate analyses (namely age at diagnosis, time to diagnosis, oral steroid dose, and PTA on the affected side). Only PTA on the affected side retained its statistical significance (OR: 1.0615, 95% CI: 1.0185–1.1063, *p* = 0.005). *Conclusions*: The analysis of our data showed an association between the hearing threshold before treatment and the recovery from ISSNHL. Further studies on larger cohorts (especially in a prospective setting) are needed to shed more light on the prognostic role of clinical parameters in patients with ISSNHL. In a correct counseling setting, with regard to the patient’s concern about not being able to recover hearing, it is important to offer perspectives of appropriate hearing rehabilitation approaches.

## 1. Introduction

Sudden sensorineural hearing loss (SSNHL) is defined as a loss of 30 decibels (dB) or more over at least three contiguous audiometric frequencies, occurring within 72 h [1]. The pathogenesis of SSNHL remains unclear, with hypotheses pointing to viral infections, vascular factors (thrombosis, embolism, or vasospasm), cochlear membrane rupture, autoimmune, metabolic, and neoplastic disorders [2]. Only 10–15% of cases have known causes [3]. Genetic association studies on SSNHL have predominantly focused on proinflammatory or prothrombotic variants in candidate genes; evidence supporting a genetic contribution to SSNHL was limited [3]. Idiopathic SSNHL (ISSNHL) is defined as SSNHL with no identifiable cause despite adequate investigation [4]. With a worldwide incidence estimated at 8–15 per 100,000 population a year, ISSHL is a common otologic emergency, accounting for 1% of all cases of sensorineural hearing loss [5].

There is a dearth of information on factors able to predict the response to treatment for ISSNHL and hearing recovery; nevertheless, different hypotheses have been proposed over time [6]. From a clinical perspective, the role played by different comorbidities has been investigated: patients with metabolic syndrome and/or diabetes mellitus, hypertension, nephropathy, and autoimmune diseases have a higher risk of ISSNHL and a poor prognosis after the occurrence of this disorder [7,8,9]. However, while none of these conditions appeared to be specifically associated with the risk of ISSNHL and the rate of hearing recovery after ISSNHL [8,9], it must be considered that it can be challenging to determine the exact impact of each disease on ISSNHL and its prognosis, as these diseases often occur in comorbidities. Consequently, multiple factors may influence ISSNHL occurrence and prognosis. From an audiological perspective, a correlation has also been demonstrated between the type and severity of hearing loss and prognosis after ISSNHL [10]. It has been shown that patients with a moderate to severe degree of hearing loss, especially if it extends across all frequencies, were at a higher risk of ISSHL and may have a poorer prognosis [11]. Although a growing body of literature exists in the field of circulatory biomarkers identification in SSNHL, there is a high heterogeneity of results and low quality of evidence [6].

The aim of this study was to apply univariate and multivariate statistical models in a retrospective clinical setting of patients given therapy for ISSNHL at our tertiary academic audiological centers to investigate the prognostic value of clinical signs, symptoms, and comorbidities in relation to hearing recovery.

## 2. Materials and Methods

### 2.1. Patients

The study was conducted according to the principles of the Helsinki Declaration, and its design was approved by the Ethical Committee of the Province of Treviso, Italy (n° 1344/CE Marca, 25 May 2023). Data were examined in agreement with Italian privacy and sensitive data laws.

A multi-center series of patients admitted to the Phoniatrics and Audiology Unit of the University of Padova and the Audiology Unit of the University of Modena and Reggio Emilia was retrospectively evaluated.

The inclusion criteria were: (i) history of ISSNHL, defined as sensorineural hearing loss of at least 30 dB, affecting 3 or more consecutive frequencies, within a 3-day time frame [4] and treated at the Padova or Modena tertiary academic audiological centers; (ii) age ≥ 18 years; (iii) availability of clinical and audiological outcome data, including complete medical history, pure-tone audiometry, speech audiometry, tympanometry, stapedial reflex, treatment details, imaging (temporal bone CT and brain MRI),

The exclusion criteria were (i) hearing loss in acoustic schwannoma, endolymphatic hydrops, meningitis, trauma (head trauma, temporal bone fracture, acoustic trauma), barotrauma, perilymphatic fistula; (ii) exposure to noise levels ≥ 80 dB in the work environment; (iii) any unilateral or bilateral hearing loss (except for presbycusis) prior to the diagnosis of ISSNHL; (iv) any disorders affecting the external or middle ear; (v) any previous ear surgery; (vi) refusal to make medical data available for research purposes.

Demographics, as well as clinical data on presenting symptoms (such as subjective hypoacusis, fullness, otalgia, tinnitus, concomitant flu syndrome, vestibular signs and symptoms), systemic comorbidities (such as diabetes, hypertension, vascular diseases, and autoimmunity), hearing thresholds at diagnosis and after treatment, and, if available, second-level diagnostics (including acoustic brainstem responses, temporal bone CT, and/or cerebellopontine angle MRI) were retrieved from patients’ charts.

Audio-vestibular symptoms and signs were defined as follows: (i) subjective hypoacusis (a decrease in auditory performance felt by the patient, compared to his/her usual hearing status); (ii) fullness (a subjective feeling of ear filling or pressure); (iii) vestibular symptoms, including vertigo (defined as an illusion of spinning motion of the surrounding environment or self-motion), and disequilibrium (a sense of imbalance, unsteadiness or wobbliness, occurring mostly when walking) [12,13]; (iv) presence of spontaneous or positional nystagmus at physical examination; (v) presence of latero-deviation; (vi) presence of asymmetric vestibular reflectivity at caloric stimulation test.

The database for archiving the data was the same in both audiological centers involved.

### 2.2. Diagnostic and Therapeutic Work-Up

At diagnosis, each patient underwent an audiological study including (i) pure-tone audiometry on the 250–500–1000–2000–4000–8000 Hz frequencies by both air and bone conduction, (ii) speech audiometry, and (iii) tympanometry with stapedial reflex measurement. The pure-tone average (PTA) was estimated as the mean value of the 500–1000–2000–4000 Hz thresholds. From speech audiometry curves, the following thresholds were considered: (i) detection threshold, (ii) 50% speech discrimination score threshold (SDS50), and (iii) 100% speech discrimination score threshold.

Second-level tests included Auditory Brainstem Response (ABR), contrast-enhanced cerebellopontine angle MRI, temporal bone CT scan, and blood tests (white blood cell count [WBC], hemoglobin [Hb], platelets’ [PLT], neutrophils’, lymphocytes’, monocytes’ counts, C-reactive protein [CRP] and erythrocyte sedimentation rate [ESR]).

In all patients, treatment was initiated as soon as the baseline hearing status had been defined, so the time to diagnosis corresponded to the treatment delay. A systemic steroid therapy was administered to all patients, either orally with prednisone (0.5 to 1 mg/kg) or intravenously with methylprednisolone (0.5 to 1 mg/kg). When clinically indicated, steroid therapy was associated with other drugs, including multi-vitaminic complexes and, in the case of vertigo, betahistine. Second-line therapy included intratympanic steroid injections (a course of 3 intratympanic injections of 0.5–1 cc of dexamethasone 10 mg/mL, over 10 days) and/or hyperbaric oxygen therapy (HBOT) (at least 20 sessions of 40 min, with an intervening 5-min air brake to prevent oxygen toxicity, under 100% O_2_ at a constant pressure of 2.2 atmospheres).

For a detailed breakdown of the therapeutic strategies employed, see Table A1 and the Results section.

The median follow-up time was 4.00 weeks (IQR: 2.00–8.00 weeks). The definition of functional outcomes was based on the modified Siegel’s criteria [14] as follows:

- Complete recovery (CR): Final hearing level ≤ 25 dB;
- Partial recovery (PR): More than 15 dB hearing gain and final hearing level 26–45 dB;
- Slight improvement (SI): More than 15 dB hearing gain and final hearing level 46–75 dB;
- No improvement (NI): Less than 15 dB hearing gain or final hearing level 76–90 dB;
- Non-serviceable ear (NS): Final hearing level > 90 dB. For the statistical analysis, the outcomes were dichotomized as CR vs. non-complete recovery (including PR, SI, NI, and NS).

### 2.3. Statistical Analysis

Continuous variables were summarized by median and interquartile range. Categorical variables were described as count and percentage in each category. The normality of continuous variables was inspected with a Q-Q plot and the Shapiro–Wilk test. The Mann–Whitney test was used to compare the distribution of continuous variables, while Fisher’s exact test was used for categorical variables. A pairwise comparison of continuous variables was performed using a two-sided sign test.

The association between continuous clinical variables and the dichotomic outcome (CR vs. non-complete recovery) was investigated with a logistic regression model. The output of such a statistical model was expressed as the odds ratio of non-complete recovery. The goodness of fit of the logistic regression model was investigated through Hosmer and Lemeshow’s test and Receiver Operating Characteristic (ROC) analysis. The probability of non-complete recovery for each variable’s value was assessed by logistic regression post-estimation tools. The logistic regression model predicted the probability of non-complete recovery for each observation based on the values of the considered independent variables. A prediction was considered “positive” if the probability of the outcome was ≥50%; otherwise, it was “negative”. The correct classification rate was defined as the proportion of cases in which the prediction and actual outcome matched (cases classified as “positive” who actually showed non-complete recovery or those deemed as “negative” presenting complete recovery).

A multivariate logistic regression model was implemented considering the non-multicollinear variables that showed a *p*-value ≤ 0.10 in the univariate analysis. Multicollinearity was tested via the computation of the uncentered variance inflation factors (uncentered VIFs). An uncentered VIF value ≥ 10 was assumed to indicate high multicollinearity risk.

Statistical significance was set at *p*-value < 0.05. Statistical analyses were performed using Stata 16.1 (College Station, TX, USA).

## 3. Results

### 3.1. General Clinical Features and Outcomes

According to the inclusion/exclusion criteria, 86 patients (38 females, 48 males; median age: 58.00 years (IQR: 47.00–69.00) were included in this retrospective study. The median time elapsed from symptom onset to clinical evaluation was 7.00 days (IQR: 1.50–20.00 days).

The distribution of demographics, clinical features, and comorbidities, as well as hearing thresholds at diagnosis, has been summarized in Table A1. The median PTA (500 to 2000 Hz) at diagnosis was 48.13 dB (IQR: 36.25–73.75 dB) on the affected side and 21.25 dB (15.0–35.00 dB) on the healthy one.

At diagnosis, 82 patients complained of subjective hypoacusis, 37 with fullness, 55 with tinnitus, 4 with otalgia, and 26 with vestibular symptoms. Regarding vestibular signs, 6 patients had spontaneous and 7 positional nystagmus, while 6 showed asymmetric vestibular reflectivity at the caloric stimulation test.

Out of 85 patients with available data on therapy, 83 received a first-line oral steroid treatment, starting with a median dose of 30.00 mg of Prednisone (IQR: 25.00–50.00 mg). Ten patients received intravenous steroids (two as upfront treatment, eight as a second-line therapy after oral steroid administration), starting with a median methylprednisolone dose of 60.00 mg (IQR: 60.00–60.00 mg). Intratympanic steroid injection was administered as a second-line therapy in two cases after an upfront oral treatment.

Forty-five patients also received concomitant therapy with other drugs (including multi-vitamin complexes and, in cases of vertigo, betahistine). In 21 cases, hyperbaric oxygen therapy (HBOT) was performed as a second-line treatment.

After a median follow-up time of 4.00 weeks (IQR: 2.00–8.00 weeks), the median PTA (500 to 2000 Hz) on the affected size significantly improved, compared to that at diagnosis (two-sided sign test: *p* < 0.001), reaching 37.50 dB (IQR: 25.00–60.00). Twenty-one (24.42%) out of 86 patients showed complete recovery, according to the modified Siegel’s criteria.

### 3.2. Clinical Prognostic Factors

Patients with diabetes showed significantly higher median PTA (500 to 2000 Hz) values at their last follow-up (Mann–Whitney U test, *p* = 0.0203) compared to those without this comorbidity. Instead, no significant differences in terms of PTA (500 to 2000 Hz) at the last follow-up were found when stratifying the included population by the presence or absence of hypertension, vascular diseases, kidney failure, dyslipidemia, and autoimmunity (one-sided Fisher’s exact test, *p* = 0.2183, *p* = 0.3152, *p* = 0.2118, *p* = 0.0879, *p* = 0.0887, respectively). Patients with diabetes also showed a higher rate of non-complete recovery, according to the modified Siegel’s criteria, compared to the non-diabetic ones (one-sided Fisher’s exact test, *p* = 0.048).

Regarding audio-vestibular symptoms and signs, subjective hypoacusis, fullness, tinnitus, and otalgia were not significantly associated with non-complete recovery risk (one-sided Fisher’s exact test, *p* = 0.560, 0.560, 0.333, and 0.454), while patients with vestibular symptoms showed a higher rate of non-complete recovery (one-sided Fisher’s exact test, *p* = 0.043). On the other hand, the presence of spontaneous or positional nystagmus, as well as asymmetric vestibular reflectivity at caloric stimulation test, were not significantly associated with non-complete recovery risk (one-sided Fisher’s exact test, *p* = 0.485, *p* = 0.370, *p* = 0.417).

Table A1 summarizes the association between clinical features and risk of non-complete recovery.

### 3.3. Prognostic Value of Audiometric Findings at Diagnosis: Logistic Regression-Based Estimations

According to the univariate logistic regression model, a significant association was found between PTA (500 to 2000 Hz) on the affected side at diagnosis and risk of non-complete recovery (OR: 1.0715, 95% CI: 1.0282–1.1167; *p* = 0.001).

**Figure 1:**
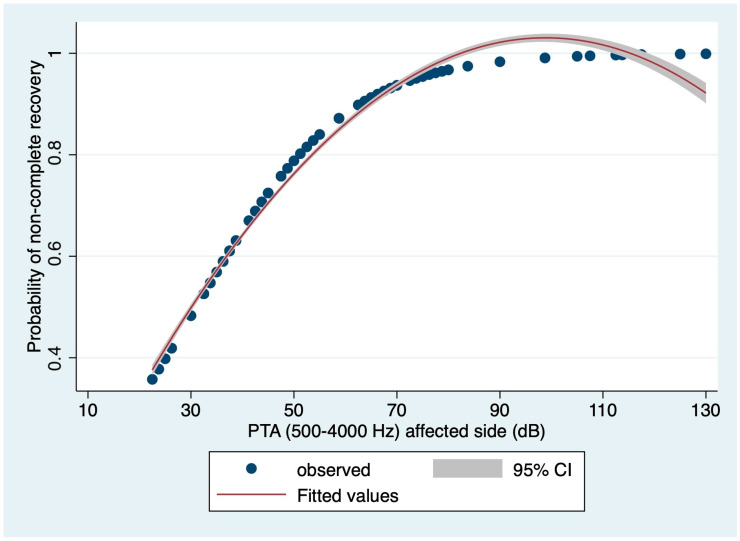
Relationship between non-complete recovery probability and PTA (500 to 2000 Hz) values on the affected side, based on the logistic regression model (dots: observations; red curve: fitted values; gray range: 95% confidence interval).

The logistic regression model based on the PTA (500 to 2000 Hz) on the affected side showed an 80.23% correct classification rate. The area under the ROC curve for this model was 0.8117, and the Hosmer and Lemeshow’s goodness-of-fit test could not reject the model (*p* = 0.1811). The relationship between non-complete recovery probability and PTA (500 to 2000 Hz) values on the affected side, based on the logistic regression model, is depicted in Figure 1.

Considering each tested frequency on the affected side, an association with the risk of non-complete recovery was significant for audiometric thresholds at 250 Hz (OR: 1.0346, 95% CI: 1.0100–1.0609; *p* = 0.008), 500 Hz (OR: 1.0388, 95% CI: 1.0126–1.0658; *p* = 0.004), 1000 Hz (OR: 1.0375, 95% CI: 1.0128–1.0628; *p* = 0.003), 2000 Hz (OR: 1.0475, 95% CI: 1.0197–1.0761; *p* = 0.001), 4000 Hz (OR: 1.0388, 95% CI: 1.0144–1.0638; *p* = 0.002), and 8000 Hz (OR: 1.0333, 95% CI: 1.0124–1.0545; *p* = 0.002).

**Figure 2:**
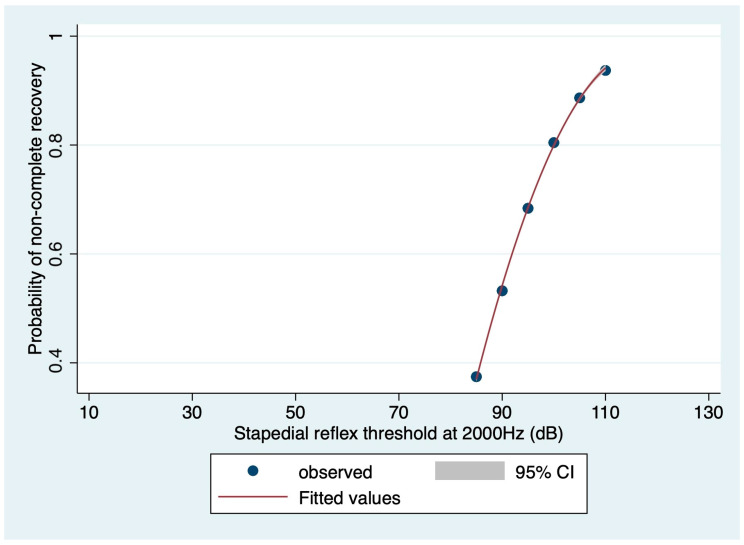
Relationship between non-complete recovery probability and the stapedial reflex threshold at 2000 Hz, based on the logistic regression model (dots: observations; red curve: fitted values; gray range: 95% confidence interval).

Regarding the prognostic value of stapedial reflex thresholds on the affected side, they appeared to be significant only at 2000 Hz (OR: 1.1371, 95% CI: 1.0087–1.2818; *p* = 0.036). The logistic regression model based on the stapedial reflex threshold at 2000 Hz showed an 80.65% correct classification rate. The area under the ROC curve for this model was 0.4057, and the Hosmer and Lemeshow’s goodness-of-fit test could not reject the model (*p* = 0.3460). The relationship between non-complete recovery probability and stapedial reflex threshold at 2000 Hz, based on the logistic regression model, is depicted in Figure 2.

**Figure 3:**
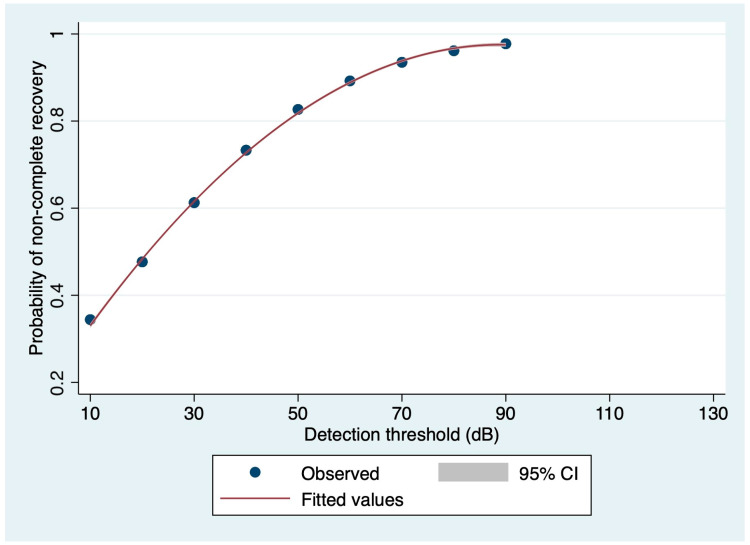
Relationship between non-complete recovery probability and detection threshold at speech audiometry, based on the logistic regression model (dots: observations; red curve: fitted values; gray range: 95% confidence interval).

Considering speech audiometry parameters from the affected side, a significant prognostic value in terms of risk of non-complete recovery was found for the detection threshold (OR: 1.0567, 95% CI: 1.0125–1.1029; *p* = 0.011; correct classification rate: 72.55%; see also Figure 3). The area under the ROC curve was 0.7759, and the Hosmer and Lemeshow’s goodness-of-fit test (*p* = 0.4257) allowed this logistic regression model not to be rejected. Instead, the association between the risk of non-complete recovery and both SDS50 and SDS100 was not significant (*p* = 0.051 and *p* = 0.202, respectively).

A multivariate logistic regression model was implemented, including the non-multicollinear clinical and audiological variables, which showed a *p*-value ≤ 0.10 at the univariate analyses (namely age at diagnosis, time to diagnosis, oral steroid dose, and PTA on the affected side). Also, at multivariate analysis, the PTA on the affected side retained its statistical significance (OR: 1.0615, 95% CI: 1.0185–1.1063, *p* = 0.005), while age at diagnosis, time to diagnosis, and oral steroid dose seemed not to independently predict hearing outcome (*p* = 0.490, *p* = 0.168, and *p* = 0.469, respectively, see also Table 1). The correct classification rate for the multivariate model was 81.48%, and the area under the ROC curve was 0.8381. The Hosmer and Lemeshow’s goodness-of-fit test could not reject the model (*p* = 0.5616).

## 4. Discussion

ISSHL is a common clinical finding for audiologists and otolaryngologists. Most patients complaining of hearing loss ask if a complete recovery is possible and what their chances are of returning to their previous hearing threshold. Such situations demand prognostic tools able to predict hearing recovery. Most of the available literature dealing with ISSHL addresses the problem of its appropriate treatment, while few investigations have looked into the clinical factors influencing the likelihood of patients’ hearing being restored [5,15].

In the present study, we aimed to investigate, by univariate and multivariate statistical models, the prognostic value of clinical signs, symptoms, and comorbidities in relation to hearing recovery using a quantitative approach.

### 4.1. Demographics, Comorbidities and Prognosis

Among demographic factors, aging (typically defined as 60 years and older in most studies) has frequently been correlated with a decrease in the rate of hearing recovery, as well as an increase in absolute hearing thresholds [16]. Since the inner ear is one of the organs with the highest mass-specific oxygen consumption, the reason for this influence of aging could be that older patients have a higher prevalence of comorbidities associated with microangiopathy, which could explain a chronic impairment of the inner ear structures and a consequently lower recovery rate in the event of an acute insult [5]. Also, in our series, the univariate statistical analysis showed that patients aged ≥65 years were significantly associated with poorer prognosis in terms of complete hearing recovery after treatment (Table A1).

The role of comorbidities in predicting hearing recovery is controversial [17]. Accompanying systemic conditions related to the incidence of ISSNHL include dyslipidemia, hypertension, coronary artery disease, cerebrovascular disease, chronic kidney disease, and anemia. However, there is an ongoing debate regarding the evidence supporting the association between these disorders and the hearing recovery rate in ISSNHL [16]. In the Askar et al. [18] investigation, diabetes mellitus was regarded as a poor prognostic indicator, in agreement with our study. A retrospective review of diabetic patients with SSNHL was conducted at National Taiwan University Hospital from 1984 to 2003 [19]. It was reported that the poor prognosis of SSNHL in diabetes patients could be caused by pre-existing microvascular lesions in the inner ear, and the post-prandial plasma glucose level could be a risk factor indicator for cochlear dysfunction in diabetic patients. Ryu et al. [20] investigated the prognostic value of hyperglycemia in predicting hearing recovery after ISSNHL. They found that the hearing recovery rate of the normal glucose tolerance (normoglycemia) group was significantly better than that of the impaired glucose regulation group; it was hypothesized that hyperglycemia might be a bad prognostic factor because it could cause microvascular damage and neuropathy. Weng et al. [19] stated that high-dose glucocorticoids should not be contraindicated in diabetic patients with SSNHL.

### 4.2. Pre-Treatment Audiological Variables and Prognosis

Our results supported the hypothesis of an association between hearing threshold before treatment and recovery from ISSNHL. According to the univariate logistic regression model, a significant association was found between PTA on the affected side at diagnosis and risk of non-complete recovery. In a multivariate logistic regression model including the non-multicollinear clinical and audiological variables, only the PTA on the affected side retained its statistical significance. Dong et al. [21] reported that the percentage of patients who achieved full recovery from SSNHL decreased as the frequency of hearing loss increased, with the lowest recovery rate in cases of high-frequency hearing loss (11.1% in the case of high-frequency hearing loss vs. 69% in the case of low-frequency hearing loss). Psillas et al. [22] found a complete hearing recovery in 77.7% of patients in the low-frequency hearing loss group and in 15% of patients in the high-frequency hearing loss one. On this issue, Choo et al. [23] reported significantly better outcomes and higher recovery rates in low-frequency than in high-frequency hearing loss. These data suggested a higher susceptibility to injury of the cochlea base, compared to the apical region, probably also due to the different vascularization of the cochlea at the apical and basal regions. It was hypothesized that there could be differences in vulnerability between the basal and apical hair cells and/or different steroid concentrations in different anatomical locations [24].

Speech perception was associated with post-treatment hearing levels, and it clearly decreased in cases of high-frequency discriminating hearing loss, particularly in noisy environments [25]. Although in a univariate statistical setting, in our series also, speech perception was found to be significantly associated with ISSNHL recovery and an indicator of poor prognosis in the case of a high threshold of speech perception. In some cases, the ability to discriminate words could be significantly worse than predicted by patients’ pure tone audiometry [26], suggesting the presence of central auditory processing impairment despite peripheral auditory function recovery.

The role of the stapedial reflex in predicting recovery rate after ISSNHL has still not been extensively established. Our results supported the hypothesis of an association between the absence of stapedial reflexes and poor prognosis, particularly at 2000 Hz. According to Margolis [27], as the amount of hearing loss increased, the likelihood of the reflex being present lessened. Consequently, the presence or absence of stapedial reflexes may be considered a potential predictor of recovery. Consistently with our findings, Gerwin and LaCoste [28] reported a significant association between stapedial reflexes and ISSNHL recovery, indicating stapedial reflexes as an accurate prognostic indicator in 32 of their 34 patients (94%).

### 4.3. Weaknesses and Strengths of the Investigation

The main limitations of this study lay in its relatively limited sample size and in its retrospective design. This made possible an a priori unique treatment schedule for all the included patients impossible and might have led to potential information biases on clinical data recall and subjective symptoms definition. However, this study’s strengths reside mostly in its multicentric setting and in the homogeneity of the considered series regarding the following aspects: (i) the inclusion/exclusion criteria were circumscribed, allowing for a substantial homogeneity in case definition; (ii) the audiological evaluation and diagnosis, based on a standardized approach, was homogeneous across the two centers; (iii) therapy was based on the AAOHNS guidelines, which recommended corticosteroids as initial therapy for ISSNHL within two weeks of symptom onset [4] (conversely, hyperbaric oxygen therapy has been suggested within one month from ISSNHL occurrence; intratympanic steroids might be used as salvage therapy in incomplete recovery after two to six weeks; other pharmacologic treatments were not routinely recommended for ISSNHL [4]); (iv) the definition of clinical outcomes was homogeneously based on the modified Siegel’s criteria.

## 5. Conclusions

ISSNHL has always been a major challenge for audiologists and otolaryngologists. Most available studies have traditionally focused on the role of ISSNHL diagnostics and pharmacological treatments. Instead, this study aimed to investigate the prognostic value of demographics, comorbidities, symptoms, and clinical signs in relation to hearing recovery using a quantitative approach. The analysis of our data showed an association between the hearing threshold before treatment and the recovery from ISSNHL. Although in a univariate setting series, patients aged ≥65 years were also significantly associated with poorer prognosis in terms of complete hearing recovery, our multivariate logistic regression model found that only PTA on the affected side retained its statistical significance.

Further studies on larger cohorts (especially in a prospective setting) are needed to shed more light on the prognostic role of clinical parameters in patients with ISSNHL. Moreover, there is a need to proceed in digging out the role of circulatory biomarkers in ISSNHL. Future well-designed investigations conducted following international guidelines, adequate collecting and reporting of biomarkers levels and using standardized methods of outcome measures could give clinicians further instruments of prognostic and therapeutic utility in ISSNHL [6].

Moreover, further clinical research (preferably in a randomized, prospective, multi-center setting) should address the safety and the effectiveness of possible alternatives to systemic steroid therapy, including intratympanic steroid administration, in particular in patients with diabetes or other conditions posing them at risk of systemic side effects.

With regard to the patient’s concern about not being able to recover hearing, in a correct counseling setting, it is important to offer perspectives of appropriate hearing rehabilitation approaches.

## Figures and Tables

**Table 1:** Results of the multivariate logistic regression analysis model, including age at diagnosis, time to diagnosis, and PTA on the affected side at diagnosis.

Variable	Odds Ratio (95% C.I.)	Standard Error	*p*-Value
Age at diagnosis	1.0174 (0.9687–1.0686)	0.0255	0.490
Time to diagnosis	1.0400 (0.9835–1.0996)	0.0296	0.168
Oral steroid dose	1.0214 (0.9646–1.0814)	0.0298	0.469
PTA on the affected side at diagnosis	1.0615 (1.0185–1.1063)	0.0224	0.005

## Data Availability

The datasets generated and analyzed during the current study are available upon reasonable request. The data are not publicly available for privacy protection.

## Appendix A

**Table A1:** Distribution of demographics, clinical features, comorbidities, and hearing thresholds at diagnosis in the whole sample and in patients who obtained complete recovery and those who did not.

Variable	Overall N = 86	Complete Recovery N = 21	Non-Complete Recovery N = 65	*p*-Value
Age (years) Median (IQR)	58.00 (47.00–69.00)	54.00 (50.00–60.00)	60.00 (46.00–63.00)	0.165 *
Age < 65 years N (%)	54 (62.79)	17 (80.95)	37 (56.92)	0.040 **
Age ≥ 65 years N (%)	32 (37.21)	4 (19.05)	28 (43.08)	
Female N (%)	38 (44.19)	11 (52.38)	27 (41.54)	0.268 **
Male N (%)	48 (55.81)	10 (47.62)	38 (58.46)	
Time-to-diagnosis (days) Median (IQR)	7.00 (1.50–20.00)	5.00 (1.00–7.00)	7.00 (2.00–20.00)	0.102 *
Time-to-diagnosis ≤ 7 days N (%)	49 (56.98)	16 (76.19)	33 (50.77)	0.035 **
Time-to-diagnosis > 7 days N (%)	37 (43.02)	5 (23.81)	32 (49.23)	
No hypertension N (%)	43 (53.09)	13 (65.00)	30 (49.18)	0.166 **
Hypertension N (%)	38 (46.91)	7 (35.00)	31 (50.82)	
No diabetes N (%)	71 (87.65)	20 (100.00)	51 (83.61)	0.048 **
Diabetes N (%)	10 (12.39)	0 (0.00)	10 (16.39)	
No vascular disease N (%)	70 (86.42)	16 (80.00)	54 (88.52)	0.268 **
Vascular disease N (%)	11 (13.58)	4 (20.00)	7 (11.48)	
No dyslipidemia N (%)	61 (75.31)	17 (85.00)	44 (72.13)	0.197 **
Dyslipidemia N (%)	20 (24.69)	3 (15.00)	17 (27.87)	
No kidney failure N (%)	79 (97.53)	20 (100.00)	59 (96.72)	0.565 **
Kidney failure N (%)	2 (2.47)	0 (0.00)	2 83.28)	
No autoimmune disorders N (%)	74 (92.50)	10 (90.00)	56 (93.33)	0.470 **
Autoimmune disorders N (%)	4 (7.50)	2 (10.00)	4 (6.67)	
No subjective hypoacusis N (%)	2 (2.38)	0 (0.00)	2 (3.17)	0.560 **
Subjective hypoacusis N (%)	82 (97.62)	21 (100.00)	61 (96.83)	
No fullness N (%)	46 (55.42)	13 (61.90)	33 (53.23)	0.333 **
Fullness N (%)	37 (44.58)	8 (38.10)	29 (46.77)	
No tinnitus N (%)	26 (32.10)	6 (28.57)	20 (33.33)	0.454 **
Tinnitus N (%)	55 (67.90)	15 (71.43)	40 (66.67)	
No vestibular symptoms N (%)	57 (68.67)	18 (85.71)	39 (62.90)	0.043 **
Vestibular symptoms N (%)	26 (31.33)	3 (14.29)	23 (37.10)	
No spontaneous nystagmus N (%)	50 (89.29)	14 (93.33)	36 (87.80)	0.485 **
Spontaneous nystagmus N (%)	6 (10.71)	1 (6.67)	5 (12.20)	
No positional nystagmus N (%)	44 (86.27)	13 (92.86)	31 (83.78)	0.370 **
Positional nystagmus N (%)	7 (13.73)	1 (7.14)	6 (16.22)	
No asymmetric vestibular reflectivity N (%)	38 (86.36)	12 (92.31)	26 (83.87)	0.417 **
Asymmetric vestibular reflectivity N (%)	6 (13.64)	1 (7.69)	5 816.13)	
Oral Steroid dose (mg) Median (IQR)	30 (25-50)	40 (25-50)	25 (25-37.5)	0.0249 *
PTA affected side (dB) Median (IQR)	48.13 (36.25–73.75)	36.25 (30.00–37.50)	58.75 (42.50–76.25)	<0.0001 *
Threshold 250 Hz affected side (dB) Median (IQR)	45.00 (30.00–65.00)	35.00 (15.00–45.00)	50.00 (35.00–70.00)	0.0038 *
Threshold 500 Hz affected side (dB) Median (IQR)	50.00 (35.00–70.00)	40.00 (25.00–45.0)	55.00 (40.00–75.00)	0.0012 *
Threshold 1000 Hz affected side (dB) Median (IQR)	50.00 (35.00–75.00)	35.00 (20.00–40.00)	60.00 (35.00–80.00)	0.0010 *
Threshold 2000 Hz affected side (dB) Median (IQR)	50.00 (30.00–75.00)	30.00 (20.00–35.00)	60.00 (40.00–75.00)	0.0001 *
Threshold 4000 Hz affected side (dB) Median (IQR)	60.00 (40–00–80.00)	45.00 (35.00–50.00)	70.00 (50.00–90.00)	0.0003 *
Threshold 8000 Hz affected side (dB) Median (IQR)	7.00 (50.00–90.00)	50.00 (40.00–65.00)	80.00 (55.00–95.00)	0.0006 *
Stapedial reflex threshold 500 Hz affected side (dB) Median (IQR)	95.00 (90.00–100.00)	95.00 (85.00–97.50)	97.50 (90.00–100.00)	0.0808 *
Stapedial reflex threshold 1000 Hz affected side (dB) Median (IQR)	95.00 (90.00–100.00)	95.00 (85.00–100.00)	100.00 (90.00–100.00)	0.1150 *
Stapedial reflex threshold 2000 Hz affected side (dB) Median (IQR)	95.00 (90.00–100.00)	87.50 (85.00–100.00)	100.00 (95.00–105.00)	0.0301 *
Stapedial reflex threshold 4000 Hz affected side (dB) Median (IQR)	95.00 (90.00–100.00)	90.00 (85.00–100.00)	100.00 (90.00–105.00)	0.1787 *
Detection threshold affected side (dB) Median (IQR)	30.00 (30.00–70.00)	30.00 (20.00–30.00)	45.00 (30.00–70.00)	0.0017 *
SDS50 affected side (dB) Median (IQR)	40.00 (30.00–50.00)	40.00 (30.00–40.00)	45.00 (40.00–55.00)	0.0323 *
SDS100 affected side (dB) Median (IQR)	50.00 (40.00–60.00)	50.00 (40.00–60.00)	50.00 (50.00–70.00)	0.2816 *

* Mann-Whitney U test; ** One-sided Fisher’s exact test.
